# Assessment of three techniques for delivering stem cells to the heart using PET and MR imaging

**DOI:** 10.1186/2191-219X-3-72

**Published:** 2013-10-28

**Authors:** Esmat Elhami, Bryson Dietz, Bo Xiang, Jixian Deng, Fei Wang, Chao Chi, Andrew L Goertzen, Shadreck Mzengeza, Darren Freed, Rakesh C Arora, Ganghong Tian

**Affiliations:** 1 Department of Physics, University of Winnipeg, 515 Portage Avenue, Winnipeg MB R3B 2E9, Canada; 2 Department of Physics & Astronomy, University of Manitoba, Winnipeg, Canada; 3 National Research Council of Canada, 435 Ellice Ave, Winnipeg MB R3B 1Y6, Canada; 4 Department of Radiology, University of Manitoba, Winnipeg, Canada; 5 Cardiac Sciences Program, Institute of Cardiovascular Sciences, St. Boniface General Hospital, 351 Tache Ave, Winnipeg, Manitoba R2H 2A6, Canada

**Keywords:** Cell tracking, Positron emission tomography (PET), Magnetic resonance imaging (MRI), Stem cell therapy, Adipose-derived stem cells, Heart failure in small animal model

## Abstract

**Background:**

Stem cell therapy has a promising potential for the curing of various degenerative diseases, including congestive heart failure (CHF). In this study, we determined the efficacy of different delivery methods for stem cell administration to the heart for the treatment of CHF. Both positron emission tomography (PET) and magnetic resonance imaging (MRI) were utilized to assess the distribution of delivered stem cells.

**Methods:**

Adipose-derived stem cells of male rats were labeled with super-paramagnetic iron oxide (SPIO) and ^18^ F-fluorodeoxyglucose (FDG). The left anterior descending coronary artery (LAD) of the female rats was occluded to induce acute ischemic myocardial injury. Immediately after the LAD occlusion, the double-labeled stem cells were injected into the ischemic myocardium (*n* = 5), left ventricle (*n* = 5), or tail vein (*n* = 4). In another group of animals (*n* = 3), the stem cells were injected directly into the infarct rim 1 week after the LAD occlusion. Whole-body PET images and MR images were acquired to determine biodistribution of the stem cells. After the imaging, the animals were euthanized and retention of the stem cells in the vital organs was determined by measuring the cDNA specific to the Y chromosome.

**Results:**

PET images showed that retention of the stem cells in the ischemic myocardium was dependent on the cell delivery method. The tail vein injection resulted in the least cell retention in the heart (1.2% ± 0.6% of total injected cells). Left ventricle injection led to 3.5% ± 0.9% cell retention and direct myocardial injection resulted in the highest rate of cell retention (14% ± 4%) in the heart. In the animals treated 1 week after the LAD occlusion, rate of cell retention in the heart was only 4.5% ±1.1%, suggesting that tissue injury has a negative impact on cell homing. In addition, there was a good agreement between the results obtained through PET-MR imaging and histochemical measurements.

**Conclusion:**

PET-MR imaging is a reliable technique for noninvasive tracking of implanted stem cells *in vivo*. Direct injection of stem cells into the myocardium is the most effective way for cell transplantation to the heart in heart failure models.

## Background

Heart failure is one of the leading causes of morbidity and mortality in developed countries [1]. A significant percentage of the heart failure patients (25% to 40%) die within the first year of diagnosis [2]. Unfortunately, heart failure cannot be cured with current therapeutic options. It is well known that cumulative loss of the viable cardiomyocytes is one of the fundamental mechanisms of heart failure. Replacement of the nonviable myocardium with new functional cardiomyocytes and vessel-constituting cells has therefore been proposed as a potential therapeutic strategy for the treatment of heart failure and myocardial infarction [3,4]. Stem cells, due to their unique potential of differentiating into various cell types, hold great promise in the treatment of various diseases including cardiac infarction, diabetes, HIV, cancer and wound healing [5,6]. Since adult stem cells have a great capacity to differentiate into a variety of cell types, cell transplantation could be an effective therapeutic strategy for the treatment of heart failure [7,8]. In this study, heart failure was used as the targeted disease to evaluate different cell delivery techniques.

Despite initial promising results obtained from preclinical studies, cell therapy has not been actualized for clinical application as a number of issues need to be fully addressed before stem cells become a routine therapeutic option. One such issue that remains to be resolved is the optimal cell delivery technique. Stem cells can be transplanted directly into a target organ and or administered peripherally via different delivery techniques (or routes). The efficacy of the different cell delivery techniques is still unclear and optimal delivery technique for a particular disease remains to be defined. Possible fates of the implanted stem cells have also not been understood [6]. Non-invasive imaging techniques have been used to evaluate potential therapeutic benefits and mechanisms of various types of stem cells [9]. Positron emission tomography (PET), with superior sensitivity, and magnetic resonance imaging (MRI), with a good spatial resolution, are considered as reliable imaging modalities for cell monitoring after implantation [10,11]. PET is able to provide information not only on cell distribution and proliferation following transplantation, but also on cell trans-differentiation. For example, [^18^ F]fluoro-2-deoxy-d-glucose (FDG), 3′-Deoxy-3′-^18^ F-fluorothymidine (^18^ F-FLT), and 9-(4-[^18^ F]fluoro-3-hydroxymethylbutyl) guanine (^18^ F-FHBG) have been used to study distribution, proliferation, and differentiation, respectively [12-15]. On the other hand, MRI is suitable for morphological and functional studies. Therefore, multimodality PET-MRI could provide more comprehensive assessment of implanted stem cells than those offered by each individual imaging technique. Thus, we used both PET and MRI in this study to monitor the implanted stem cells in the stem cell therapy of heart failure in small animal models.

In order to be visible on PET and MR images, the stem cells are labeled with a PET tracer and an MRI contrast reagent, respectively, prior to cell transplantation. In our previous study, we evaluated the effects of the labeling probes on the biological function of adipose-derived stem cells (ASCs) and we have optimized conditions for labeling stem cells with FDG and SPIO [16,17]. Adipose-derived stem cells were chosen as a representative of adult stem cells because the adipose tissue is easy to obtain and the use of ASCs is associated with fewer ethical concerns than with the use of embryonic stem cells [18,19]. In this study, histochemical analysis and molecular assays were used as adjunctive techniques to confirm imaging findings.

## Methods

The animals used in this study were treated with humane care in compliance with the Canadian Council on Animal Care Guide. The experimental protocols were approved by the National Research Council of Canada Animal Care Committee.

### Preparation of adipose-derived stem cells (ASCs)

The ASCs were prepared according to the method developed by Zuk et al. [19] with modifications as previously described [17]. In brief, subcutaneous adipose tissue (3 to 4 g) was obtained from the abdominal and inguinal regions of inbred (male) Lewis rats. The excised adipose tissue was washed extensively with phosphate-buffered saline (PBS; HyClone, Logan, UT, USA) to remove debris and blood cells. The adipose tissue was minced and digested with collagenase I (2 mg/ml, Worthington Biochemical Corp., Lakewood, NJ, USA) at 37°C for 20 to 30 min. Active collagenase was neutralized by adding Dulbecco's modified Eagle's medium (DMEM; HyClone) containing 15% fetal bovine serum (FBS; HyClone). The digested adipose tissue was filtered with a 100-μm strainer to eliminate the undigested fragments. The cellular suspension was centrifuged at 1,000 × *g* for 10 min. The cell pellets were resuspended in DMEM containing 15% FBS and cultivated for 48 h at 37°C in 5% CO_2_. Unattached cells and debris were removed and fresh medium was added to the adherent cells. The cells used for labeling analysis were at *passage 1*, and samples of 1.5 × 10^5^ to 2 × 10^5^ ASCs were prepared.

### Labeling of the adipose-derived stem cells (ASCs)

We have previously characterized viability and proliferation of stem cells labeled with FDG and SPIO nanoparticles in adipose-derived stem cells in separate studies, where optimum methods of cell labeling were developed [16,17,20].

Prior to imaging, three samples of ASCs (1.5 × 10^5^ to 2 × 10^5^ cells in 5 ml) were cultivated in a complete medium containing 20 μg/ml SPIO nanoparticles for 48 h. On the day of imaging, the stem cells were labeled with FDG, a readily available PET radiotracer. FDG was supplied by the Winnipeg Cyclotron Facility, at the Kleysen Institute for Advanced Medicine, Health Sciences Centre, Winnipeg, MB, which produces FDG for clinical use. ASCs were labeled with FDG using the protocol developed in our previous studies [16,21]. The activities of FDG in the cell samples were measured with a standard dose calibrator (Capintec CRC 10, Capintec, Inc., Ramsey, NJ, USA). As FDG is a glucose analogue, the ASCs were incubated in glucose-free medium for 30 min prior to labeling with FDG. Note that the terms ‘label’ and ‘FDG’ are used interchangeably.

The cells were labeled with FDG concentrations up to 3.70 MBq (100 μCi) per 1 ml (approximately 20 Bq/cell) for 60 min in 5 ml of glucose-free medium. After the labeling period, the medium was removed and the cells were washed with PBS twice and then incubated in complete medium for 30 min for efflux of any free FDG. The efflux medium was removed and cells were washed with PBS once. The amount of radioactivity in the removed medium and washes, in each stage, were measured using a dose calibrator and was used to determine the rate of FDG retention by the cells.

The cells were then trypsinized and centrifuged for 5 min at 1,000 × *g*. Cell concentration and viability was measured using the trypan-blue (0.4%) exclusion assay. From the cell suspension, depending on the method of injection, volumes of 0.4 to 0.7 μl were prepared for injection into the animal subjects. The radioactivity of the labeled cells and the syringe residual were measured before and after injection to determine the amount of the activity injected. All data were corrected for ^18^ F physical decay.

### Animal models and cell delivery methods

The animal models of left anterior descending coronary artery legation (i.e., heart failure) were created either on the day of imaging or 1 week prior to imaging. Inbred (female) Lewis rats were anesthetized with 2% isoflurane and the respiration rate controlled with a rodent ventilator. A 3-cm transverse incision was made between the fourth and fifth intercostal spaces to expose the heart. The left anterior descending coronary artery (LAD) of the heart was then occluded to induce acute ischemic myocardial injury. Immediately after the LAD occlusion, the double-labeled ASC were transplanted into the animals via one of three methods: (a) direct injection into ischemic heart muscle (*n* = 5), (b) injection into the left ventricle (*n* = 5), and (c) injection into a tail vein (*n* = 4). On average, the numbers of cells and FDG activity injected for each group were: (a) (4.5 ± 1.6) × 10^6^ ASCs, 1.0 ± 0.7 MBq FDG; (b) (5.5 ± 1.9) × 10^6^ ASCs, 1.45 ± 0.77 MBq FDG; and (c) (6.0 ± 0.8) × 10^6^ ASCs, 3.4 ± 2.6 MBq FDG. The experimental details are summarized in Table 1. In order to examine the effect of sub-acute myocardial infarction on the retention of the stem cells in the heart region, a group of animals (*n* = 3) were injected with labeled stem cells directly into the myocardium 1 week after LAD occlusion. On average, (6.3 ± 0.6) × 10^6^ ASCs, 1.7 ± 1.2 MBq FDG were injected.

**Table 1:** Summary of animal studies

**Number**	**Weight (g)**	**Model**	**Injection site**	**No. of stem cells injected**	**FDG activity (MBq)**	**Notes**
1	448	Infarcted	Myocardium	4.4 million	0.44	
2	242	Infarcted	Myocardium	4 million	0.41	
3	215	Infarcted	Myocardium	6.5 million	1.78	
4	278	Infarcted	Myocardium	2.3 million	0.63	
5	279	Infarcted	Myocardium	5.4 million	1.70	
1	238	1 week infarcted	Myocardium	6.8 million	2.22	
2	236	1 week infarcted	Myocardium	6.6 million	0.41	
3	226	1 week infarcted	Myocardium	5.6 million	2.60	
1	290	Infarcted	Left ventricle	7 million	1.63	The animal died 1.5 h after injection
2	238	Infarcted	Left ventricle	6 million	1.37	The animal died just before the start of the scan
3	203	Infarcted	Left ventricle	6.6 million	2.44	Animal survived
4	233	Infarcted	Left ventricle	3.7 million	1.52	Animal died before the whole-body scan
5	218	Infarcted	Left ventricle	4 million	0.30	Animal died during the whole-body scan
1	226	Infarcted	Femoral vein	5.4 million	2.44	
2	200	Infarcted	Femoral vein	6.3 million	0.90	
3	231	Infarcted	Tail vein	5.3 million	3.92	
4	260	Infarcted	Tail vein	7 million	7.03	

The animal models were injected with double (FDG-SPIO)-labeled ASCs via different methods of cell delivery. Each group of studies is numbered. All animals were imaged with microPET and 7-T MRI scanners. Whole-body PET data were acquired following the images of the heart at the center of field of view. The animals that died during the PET imaging (in the left ventricle injection group) were not imaged with MR.

Following cell transplantation, the animals were placed in prone position on a custom-designed cradle that was compatible with PET and MR imaging scanners and contained fiducial markers, filled with FDG and CuSO_4_ (an MR contrast) for coregistration of the PET and MR images. The amount of FDG in the fiducial markers was approximately 0.1% of the activity injected so that their intensity in the image would not interfere with that of the subject under study.

### PET imaging

A microPET P4 dedicated animal PET system (Siemens Preclinical Solutions, Knoxville, TN, USA) was used in this study. Briefly, the system has an axial field of view (FOV) of 7.8 cm and a translational FOV of 19 cm, with a spatial resolution of 1.8 mm at the center of the FOV [22]. Prior to imaging, the system was calibrated by imaging a rat-sized cylinder phantom filled with a known concentration of FDG. Immediately after cell transplantation, PET emission images of the thorax were acquired for 30 min, with the heart at the center of FOV. It was followed by whole-body imaging using three bed positions, with 10-min emission scan per bed. Corresponding transmission scans for each bed position were acquired using a ^57^Co point source, to be used for attenuation and scattering corrections.

PET data were reconstructed using the iterative OSEM3D/MAP algorithm provided by the system manufacturer, with all available corrections applied, including that for attenuation and scattering. The images were analyzed using the software ASIPro VM™ ver. 6.3.3.0 (Siemens Preclinical Solutions, USA) to quantify the biodistribution of FDG-labeled stem cells. The percentage of injected dose (ID%) in various organs was calculated for each injection method and imaging time point. The organs of interest included the heart, lungs, brain, kidneys, and bladder. Volumes of interest (VOIs) were drawn on the transverse planes (slice thickness 1.2 mm) and repeated to cover the whole organ, allowing the total activities to be determined. The total activity (in MBq or μCi) in each organ was determined using the calibration factor determined from the cylinder scan and divided by the total injected activity to determine the final percentage of injected dose for each organ. All activities were corrected for ^18^ F physical decay.

### MR imaging

After the PET imaging, the animal was moved to a 7-T MRI scanner in the same cradle. Transverse MR images were acquired for the body region containing the heart. Coronal images covering the whole body of the animal were acquired. MR images were acquired using a FLASH sequence with 1.43 ms of echo time (TE), 4.6 ms of repetition time (TR), and 17.5° of Gaussian excitation pulse. The sequence was ECG-triggered and respiratory gated. The number of subjects and PET-MR imaging are summarized in Table 1.

### Quantitative real time polymerase chain reaction (qRT-PCR)

After the imaging sessions, the animals were euthanized and vital organs were harvested and preserved in liquid nitrogen for measurement of the implanted stem cells. To do so, DNA from the organ samples was first extracted. The DNA samples and SYBR® Green PCR Kit (Cat#: 204052, Qiagen Inc., Mississauga, ON, Canada) was applied onto a PCR machine (AB 7500, Applied Biosystems, Foster City, CA, USA). A specific sequence of rat *Sry3* gene in the Y chromosome was chosen as a target gene. The primer pairs for *Sry3* are 5′-GTT CAG CCC TAC AGC CTG AGG ACA T-3′ and 5′-GGA TTC TGT TGA GCC AAC TTG CGC C-3′. The cycling conditions were 5 min at 95°C for the activation of polymerase, then 10 s at 95°C for denaturation, and 30 s at 60°C for annealing and extending. Forty cycles were used.

Genomic DNA extracted from a few male ASC samples with known numbers of cells were used to establish a standard curve to correlate the number of cells and the number of cycles at which the fluorescence exceeds the threshold; the latter is often abbreviated Ct. An excellent correlation (*r* = 0.99) was found between the number of male ASCs and Ct. The number of the implanted ASCs retained in the organs was estimated using the Ct measured in the tissue samples and the standard curve.

### Statistical analysis

Statistical analysis was performed using Statistica v.10.1011. The data were analyzed using the one-way ANOVA and followed by the post hoc Tukey HSD test to determine the significance between the injection methods; values less than 0.05 were considered statistically significant. Results are reported as mean ± standard deviation (SD) of the percentage of injected dose.

## Results and discussion

**Figure 1:**
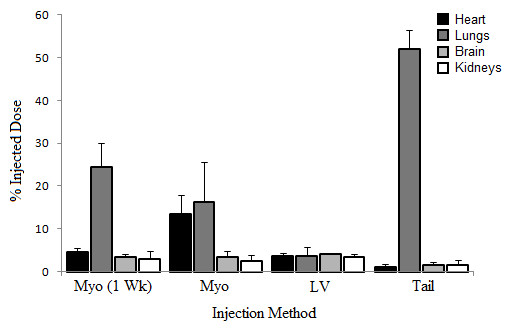
**Percentage of injected dose of FDG-labeled ASCs in various organs for different methods of injection as determined from PET data.** Percentage of injected dose values are presented as the mean value, with + SD error bars. Myo (1 Wk), direct myocardial injection after 1 week of induced infarction; Myo, direct myocardial injection; LV, left ventricle injection; Tail, tail vein injection. Refer to the text for information on the numbers of samples in each group.

**Figure 2:**
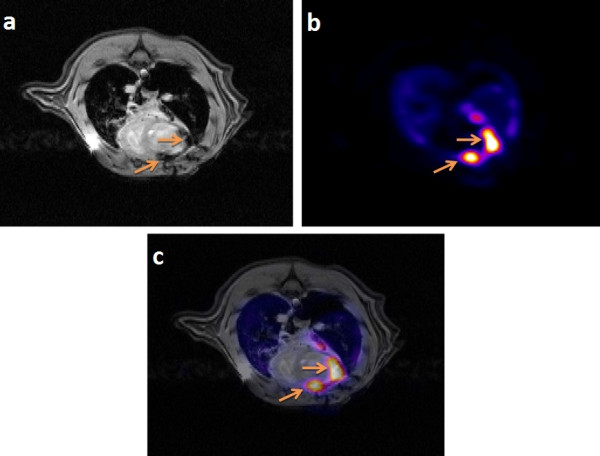
**Example of PET and MR coregistered images.** MR image **(a)**, PET image **(b),** and registered PET-MR image **(c)** of the heart of a rat injected with FDG- and SPIO-labeled stem cells directly in the myocardium, showing the high concentration of stem cells in the myocardium. The dark regions (indicated by arrows) in the MRI image are the SPIO-labeled stem cells, whereas the bright regions (indicated by arrows) in the PET image are FDG-labeled stem cells. Images were registered using MIPAV software using landmark registration algorithm. The PET-MR registered image was used to accurately define the region of interest for quantifying the ID% of the stem cell retention.

The initial biodistribution of the labeled ASCs in the heart region was determined through the VOI analysis of the first emission PET images acquired few minutes post injection of the labeled ASCs, with the heart at the center of field of view (CFOV). The concentration of the stem cells in other organs was determined through VOI analysis of the whole-body PET images, acquired at approximately 1 hour after the cell injection. Quantitative data of the PET images are summarized in Table 2. The results for individual organs for different methods of injection are summarized in Figure 1. The data represents the mean percentage of injected dose value with the standard deviation. The numbers of samples for each organ and injection method are as follows: heart - myocardium after 1 week (*n* = 2), myocardium (*n* = 4), left ventricle (*n* = 4), tail vein (*n* = 3); lungs - myocardium after 1 week (*n* = 3), myocardium (*n* = 5), left ventricle (*n* = 4), tail vein (*n* = 3); kidneys - myocardium after 1 week (*n* = 3), myocardium (*n* = 3), left ventricle (*n* = 2), tail vein (*n* = 1); the tail vein injection method had little data, as the kidneys were indistinguishable; brain - myocardium after 1 week (*n* = 2), myocardium (*n* = 3), left ventricle (*n* = 1), tail vein (*n* = 4). Figure 2 shows a representative PET-MR coregistered image, from which accurate localization of the stem cells retained in the myocardium was obtained.

**Table 2:** PET image quantification results

	**ID% ± SD (PET data)**			
	**Heart**	**Lungs**	**Brain**	**Kidneys**
Myocardial (1 week)	4.5 ± 1.1	24 ± 6	3.4 ± 0.2	2.9 ± 2.0
Myocardial	14 ± 4	16 ± 9	3.4 ± 1.5	2.5 ± 1.4
Left ventricle	3.5 ± 0.9	3.7 ± 2.0	4.15	3.3 ± 0.8
Tail vein	1.2 ± 0.6	49 ± 6	1.5 ± 0.3	1.6 ± 1.2
*p* value	0.000653	0.000022	0.054170	0.596446

The values of ID% ± SD for various organs in each injection method are presented. The *p* values were calculated using the one-way ANOVA with Statistica statistical analysis software; values less than 0.05 are considered to be statistically significant.

It was found that direct injection into the myocardium immediately after the LAD occlusion provided the largest stem cell retention in the injured myocardium (14% ± 4% of the total injected cells). Interestingly, direct injection into the infarcted myocardium 1 week after the LAD occlusion gave rise to only 4.5% ± 1.1% of the injected cells retained in the heart muscle. The cell retention in the myocardium was significantly less than that achieved with the same cell delivery technique performed immediately after LAD occlusion. This demonstrates that time of cell transplantation following a myocardial infarction also greatly affects cell retention in the targeted organ. This finding also indicates the importance of early cell transplantation. In addition, injection into the left ventricle and a tail vein led to only 3.5% ± 0.9% and 1.2% ± 0.6% of the transplanted cells, respectively, remaining in the heart muscle (Figure 1). Together, our quantitative results show that retention of the stem cells in the target region, i.e., ischemic myocardium, depends greatly on the cell delivery methods and time. Injection into the left ventricle was associated with a high mortality rate.

**Figure 3:**
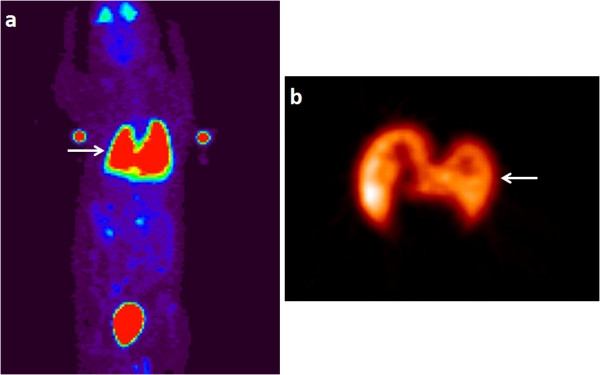
**Biodistribution of labeled ASCs in a rat injected via tail vein.** Maximum intensity projection of whole-body PET image **(a)** of a rat injected with double-labeled ASCs via tail vein (25 μg/ml SPIO and 2.3 MBq FDG). The image is reconstructed with MAP algorithm and attenuation corrected. The panel on the right **(b)** represents the image with the heart at the center of field of view. The arrow heads point out to the lungs (solid) and the heart (dashed). It is evident that the majority of the cells are retained in the lungs and the heart is not visible.

**Figure 4:**
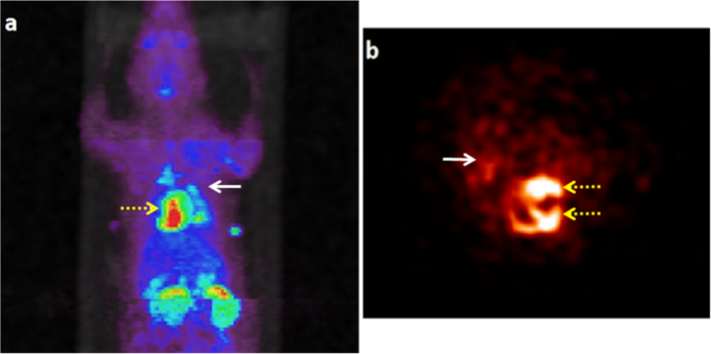
**Biodistribution of labeled ASCs in a rat injected via left ventricle.** Maximum intensity projection of whole-body PET image **(a)** from a rat injected with double-labeled ASCs via left ventricle (25 μg/ml SPIO and 1.34 MBq FDG). The image is reconstructed with MAP algorithm and attenuation corrected. The arrow heads point out to the lungs (solid) and the heart (dashed). The panel on the right **(b)** represents the image with the heart at the center of field of view. It is evident that a portion of the cells are retained in the myocardium, with some in the lungs.

**Figure 5:**
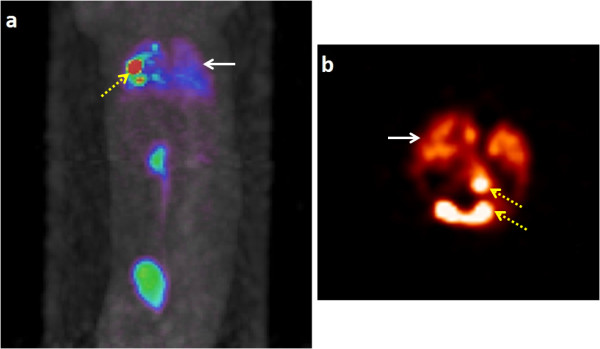
**Biodistribution of labeled ASCs in a rat injected via myocardium.** Maximum intensity projection of whole-body PET image **(a)** from a rat injected with double-labeled ASCs via myocardium (25 μg/ml SPIO and 0.44 MBq FDG). The image is reconstructed with MAP algorithm and attenuation corrected. The arrow heads point out to the lungs (solid) and the heart (dashed). The panel on the right **(b)** represents the image with the heart at the center of field of view. It is evident that the majority of the cells are retained in the myocardium, with some in the lungs.

As expected, the tail vein injection resulted in a majority of the stem cells trapped in the lungs (49% ± 6%) with negligible number of cells in the heart. This is simply due to the smaller diameter of the pulmonary capillaries relative to the size of the stem cells. A similar result was found in our previous study conducted on healthy animals [16]. In contrast, direct injection into the myocardium, either immediately or 1 week after LAD occlusion, resulted in a significantly less number of cells trapped in the lungs than the intravenous injection did. Cell retention in the lungs with the two injection times were 16% ± 9% and 24% ± 6%, respectively. The slightly higher pulmonary retention of the cells delivered 1 week after LAD ligation may be related to the injection of some cells into the right ventricle due to the thinning of the ventricular walls. The left ventricle injection yielded the lowest retention of cells in the lungs (3.7% ± 2.0% of the ID), and only one animal survived the entire PET-MR imaging. The brain and kidneys both had small percentages of cell retention with all three cell delivery methods. The bladder was found to have a radioactivity percentage of 16% ± 5%, which could be due to FDG efflux out of the labeled cells. The biodistribution of the stem cells in the body is distinctly different among the different methods of cell delivery as shown in Figures 3, 4, and 5.

**Figure 6:**
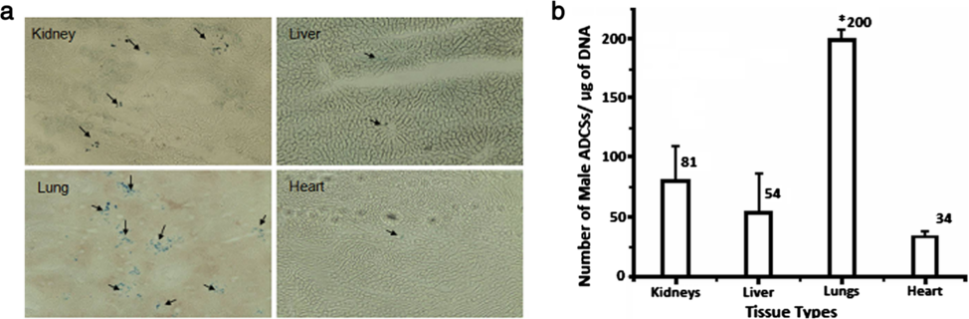
**Histochemical and PCR results for tail vein injection.** Microscopic pictures of histochemical assays **(a)**; arrows indicate Prussian blue-stained SPIO in the cells. Average number of ASCs per microgram of DNA in different tissue types of female animals injected with male ASCs via the tail vein **(b)**; each bar represents the average for *n* = 3 subjects and the error bars are + SD (*p < 0.01, *n* = 3).

**Figure 7:**
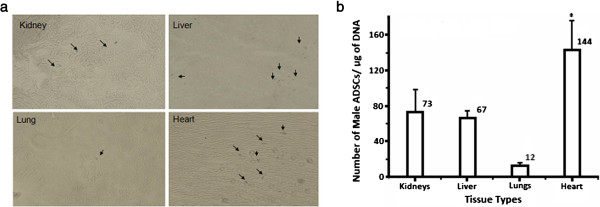
**Histochemical and PCR results for left ventricle injection.** Microscopic pictures of histochemical assays **(a)**; arrows indicate Prussian blue-stained SPIO in the cells. Average number of ASCs per microgram of DNA in different tissue types of female animals injected with male ASCs into the left ventricle **(b)**; each bar represents the average for *n* = 3 subjects and the error bars are + SD (*p < 0.01, *n* = 3).

**Figure 8:**
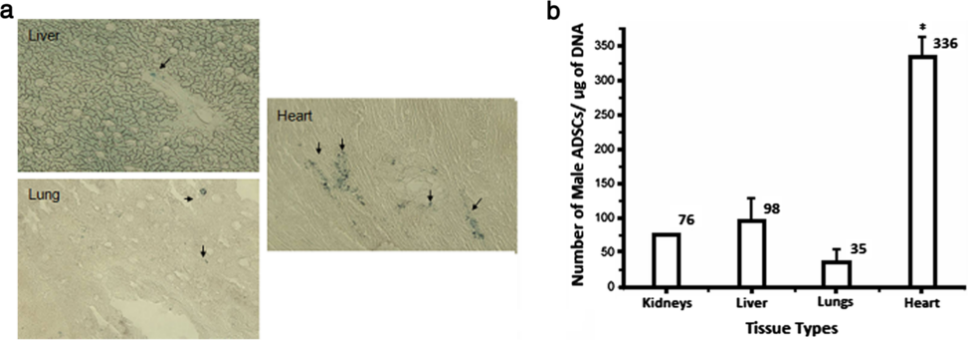
**Histochemical and PCR results for myocardial injection.** Microscopic pictures of histochemical assays **(a)**; arrows indicate Prussian blue-stained SPIO in the cells; Average number of ASCs per microgram of DNA in different tissue types of female animals injected with male ASCs into the myocardium **(b)**; each bar represents the average for *n* = 3 subjects and the error bars are + SD, kidney tissue (*n* = 1) (*p < 0.01, *n* = 3).

The results of histochemical analysis with Prussian blue staining of the SPIO nanoparticles in organ tissues are in good agreement, qualitatively, with the molecular biology approach, using the real-time polymerase chain reaction (RT-PCR). The histochemical and molecular biology data for each injection group are shown in Figures 6, 7, and 8. Cell retention in the critical organs (heart, liver, lungs, and kidneys) is expressed by the number of stem cells detected in an amount of tissue yielding 1 μg of DNA and is summarized in Table 3. The results indicate that 336 ± 28 cells were found in the myocardium with direct myocardial injection, significantly higher than the other methods of cell delivery (*p* < 0.05), while 144 ± 32 and 34 ± 5 cells were found in the intraventricular injection and intravenous injection, respectively (Table 3). As mentioned above, the number of cells retained in the lungs was highest (200 ± 8) for the tail vein injection group (*p* < 0.05), significantly higher than those found with other cell delivery methods (Table 3). The frequency of the stem cells in the kidneys and liver was comparable among all three cell delivery methods, indicating that migration of the cells to non-targeted organs is independent on the cell delivery method.

**Table 3:** PCR results

	**Number of male ADSCs per μg of DNA**			
	**Heart**	**Lungs**	**Liver**	**Kidneys**
Myocardial	336 ± 28	35 ± 20	98 ± 32	76 (*n* = 1)
Left ventricle	144 ± 32	12 ± 4	67 ± 8	73 ± 26
Tail vein	34 ± 5	200 ± 8	54 ± 33	81 ± 29
*p* value	0.000018	0.000004	0.208397	0.942587

Number of male ASCs per 1 μg of DNA tissue ± SD in various organs for each injection method (*n* = 3, except for the kidneys in the myocardial injection group, *n* = 1). The *p* value was calculated using the one-way ANOVA with Statistica statistical analysis software; values less than 0.05 are considered to be statistically significant.

The intravenous injection resulted in the lowest cell retention rate in the heart (1.2% ± 0.6%), which is consistent with results reported by Kang [23], Wolfs [24], and Boinos [25]. As mentioned above, the high retention of the implanted stem cells in the lungs is due to the fact that the majority of the stem cells are larger than the alveolar lung capillaries [26]. The low cardiac retention with the tail vein injection suggests that this method is not effective for delivery of stem cells into the myocardium. In this study, we found that direct myocardial injection yielded the highest cardiac retention of the implanted cells (14% ± 4%), relative to the other two cell delivery techniques. This finding is consistent with the results (17.8% ± 7.3%) presented in Terrovitis' study in which cardiac-derived stem cells (CDCs) were injected into rat hearts [27]. However, the cardiac retention value found in our study is slightly lower than that reported by Chan and Abraham (22.9% ± 5.2%) [26], which may be related to variation in technical skills of the operators in the studies. Nevertheless, both studies demonstrated superior cell retention with direct myocardial injection relative to other techniques. Moreover, our study showed that immediately following LAD occlusion (heart attack), direct myocardial injection offered greater cardiac cell retention relative to injection 1 week after LAD occlusion (Figure 1), indicating that timing of cell transplantation is also a critical factor in determining efficacy of cell therapy. The low cardiac retention observed in the late injection group (1 week after LAD occlusion) was probably due to the formation of scar tissue and thinning of the ventricular wall [26]. The scar tissue makes it more difficult for the stem cells to be grafted to the infarcted region and therefore fewer stem cells will be found in the heart, as indicated by our results.

There was no significant difference in radioactivity in the kidneys or brain between injection methods, indicating that the efflux throughout each injection method remains constant. The bladder yielded 16% ID, which would be entirely due to the efflux of the FDG out of the stem cells into the bloodstream. It should be noted that even though FDG can efflux from the labeled cells and accumulate in the renal system, i.e., kidneys and bladder, the PCR measurements show that stem cells were retained in the kidneys. It therefore could be implied that the FDG activity seen in the kidneys was associated with the cells, and not from free FDG. On the other hand, it is impossible to clearly distinguish between the cell-associated FDG and free FDG with the techniques used in this study. Nonetheless, a good agreement between PCR measurements and PET images found in this study validates the use of non-invasive *in vivo* PET imaging to determine the biodistribution of the stem cells throughout the body.

As mentioned above, only one out of four animals subjected to intraventricular injection survived for brain imaging. Thus, there is no error bar on the column for FDG activity in the brain on Figure 1. For the same reason, sample size for the intraventricular injection was small for the kidneys and the bladder. We believe that the high mortality rate associated with intraventricular cell injection was due to blockage of the coronary microvascular system, leading to acute diffuse myocardial ischemia. Nevertheless, this complication suggests that intraventricular injection is not a safe route for cell delivery.

This study has a couple of limitations, which will be addressed in our future studies. The short half-life of FDG limited the length of the PET imaging and cell tracking studies to 4 h following injection. Also, FDG can efflux from the stem cells at later time points, which limits the length of PET imaging and quantification to few hours post injection. Thus, FDG can only be used for quantification of the initial biodistribution of the implanted stem cells. In addition, use of fiducial markers was associated with image degradation in some of the PET data, due to the manual filling of the markers which could have resulted in FDG concentrations non-consistent with 0.1% of the injected FDG-labeled cells. Those data were not included in the data analysis. Standard markers will be a better choice in order to eliminate loss of data.

## Conclusions

In this study, we used PET and MR imaging to evaluate three methods and two time points of cell delivery in a rat model of congestive heart failure. The results of this study showed that the direct myocardial injection immediately after a heart attack (LAD occlusion in this study) resulted in the maximum cardiac retention of the implanted cells, followed by left ventricle injection. However, the latter method resulted in a high mortality rate. The tail vein injection resulted in the least percentage of cells retained in the heart, with most of them trapped in the lungs. Therefore, direct myocardial injection is the most effective method for the delivery of stem cells to the myocardium. The results of the quantification of cell retention in various organs using PET image data were validated with *ex vivo* PCR analysis in which the numbers of stem cells in various tissues were measured. This study showed that PET in combination with MR imaging is a reliable non-invasive tool in quantifying the biodistribution of the implanted stem cells *in vivo*.

## Abbreviations

ASCs: Adipose-derived stem cells; PET: Positron emission tomography; MRI: Magnetic resonance imaging; 18 F-FDG: [^18^ F]fluoro-2-deoxy-d-glucose; SPIO: Superparamagnetic iron oxide.

## Competing interests

The authors declare that they have no competing interests.

## Authors’ contributions

EE is the corresponding author. She designed, drafted, and revised the manuscript, coordinated the experiments, carried out the PET imaging data acquisition, and was involved in PET data analysis and results (figures, tables). BD was involved in PET image data acquisition and stem cell labeling with FDG, carried out PET image analysis, developed PET-MER image registration software, and created the figures and tables. BX and JD carried out the wet-lab work, preparation and labeling of the stem cells with FDG and SPIO, PCR measurements, data analysis, histochemical analysis, and provision of microscopic pictures. BX also developed and carried out MR imaging sequences and supervised the wet-lab studies. FW and CC handled the animal models, carried out surgeries to create the heart failure models, the stem cell injection, and provided animal care. AG provided the PET scanner and the financial support and guidance in the design of the experiment, as well as the insightful and important revisions to the manuscript. SM provided the FDG, radiochemistry support, advice on the experimental design, and revisions to the manuscript. DF provided advice on the experimental design. RA provided insightful advice on the experimental design, revisions to the manuscript, and general knowledge contribution to the stem cell studies. GT, as the senior supervisor of the studies, provided the financial support for the experiments through his CHIR Team Grant, design of the experiments, and materials and laboratory resources and did the revisions of the manuscript. All authors read and approved the final manuscript.
